# Zinc agronomic biofortification of staple crops may be a cost-effective strategy to alleviate zinc deficiency in Ethiopia

**DOI:** 10.3389/fnut.2022.1037161

**Published:** 2022-11-10

**Authors:** Abdu Oumer Abdu, Hugo De Groote, Edward J. M. Joy, Diriba B. Kumssa, Martin R. Broadley, Dawd Gashu

**Affiliations:** ^1^Center for Food Science and Nutrition, Addis Ababa University, Addis Ababa, Ethiopia; ^2^Sustainable Agrifood Systems Program, International Maize and Wheat Improvement Center (CIMMYT), Nairobi, Kenya; ^3^Faculty of Epidemiology and Population Health, London School of Hygiene and Tropical Medicine, London, United Kingdom; ^4^Rothamsted Research, West Common, Harpenden, United Kingdom; ^5^School of Biosciences, University of Nottingham, Loughborough, United Kingdom

**Keywords:** biofortification, DALYs, zinc, Zn-enriched fertilizers, Ethiopia

## Abstract

**Background:**

Inadequate dietary zinc (Zn) supplies and Zn deficiency (ZnD) are prevalent in Ethiopia, where cereals are major dietary sources, yet low in bioavailable Zn. Zinc agronomic biofortification (ZAB) of staple crops through application of Zn fertilizers may contribute to alleviating ZnD. However, large-scale promotion and adoption of ZAB requires evidence of the feasibility and public health benefits. This paper aimed to quantify the potential cost-effectiveness of ZAB of staple crops for alleviating ZnD in Ethiopia.

**Methods:**

Current burden of ZnD among children in Ethiopia was quantified using a disability-adjusted life years (DALYs) framework. Evidence on baseline dietary Zn intake, cereal consumption, and fertilizer response ratio was compiled from existing literature and secondary data sources. Reduction in the burden of ZnD attributable to ZAB of three staple cereals (maize, teff, and wheat) *via* granular and foliar Zn fertilizer applications was calculated under optimistic and pessimistic scenarios. The associated costs for fertilizer, labor, and equipment were estimated in proportion to the cropping area and compared against DALYs saved and the national Gross Domestic Product capita^–1^.

**Results:**

An estimated 0.55 million DALYs are lost annually due to ZnD, mainly due to ZnD-related mortality (91%). The ZAB of staple cereals *via* granular Zn fertilizer could reduce the burden of ZnD by 29 and 38% under pessimistic and optimistic scenarios, respectively; the respective values for ZAB *via* foliar application were 32 and 40%. The ZAB of staple cereals *via* granular fertilizer costs US$502 and US$505 to avert each DALY lost under optimistic and pessimistic scenarios, respectively; the respective values for ZAB *via* foliar application were US$226 and US$ 496. Foliar Zn application in combination with existing pesticide use could reduce costs to US$260–353 for each DALY saved. Overall, ZAB of teff and wheat were found to be more cost-effective in addressing ZnD compared to maize, which is less responsive to Zn fertilizer.

**Conclusion:**

ZAB of staple crops *via* granular or foliar applications could be a cost-effective strategy to address ZnD, which can be integrated with the existing fertilizer scheme and pesticide use to minimize the associated costs.

## Introduction

An estimated one-third of the global population is affected by one or more micronutrient deficiencies, including zinc deficiency (ZnD) (1), with the highest prevalence in sub-Saharan Africa (2). Globally, ZnD contributes to an estimated half a million child and infant deaths annually (1.5% of the global disease burden) attributable to diarrhea and pneumonia (3). In sub-Saharan Africa, an estimated 51–99% of adults have inadequate dietary Zn intakes, while an estimated 32–63% of children are Zn deficient (4). In Ethiopia, ZnD affects an estimated 30–51% of children (5–7) and a recent national estimate showed that ZnD affects 72% of the population (8). Soil ZnD is also widespread (9), affecting more than 70% of the soils in the highlands of Ethiopia (10), and this is likely to impair yields and Zn concentrations in staple crops for human consumption. Improved soil fertility management, including, where appropriate, use of Zn-enriched fertilizers, might contribute to better soil fertility and nutritionally enhanced crop composition (11, 12).

Agronomic biofortification is the process of increasing micronutrient concentration in the edible portion of crops through crop management strategies, including the application of micronutrient fertilizers (13, 14). Mineral micronutrients can be incorporated into granular fertilizers applied to the soil, or to the leaves as foliar sprays. It may complement other interventions to alleviate micronutrient deficiencies (15), in particular through reaching rural populations in resource-limited settings where there is limited access to animal-source foods or consumption of fortified foods. It can improve the nutritional quality of crops and provide a public health benefit with minimal risk to health (16). Low concentrations of several minerals in staple cereals are spatially correlated with child mortality in sub-Saharan Africa, although the potential of ZAB to improve nutritional status and decrease child mortality may depend on multiple factors, including the prevalence of malaria (17).

In field trials, application of Zn fertilizers increased the concentration of Zn in staple cereal grains (18, 19) and likely increased bioavailability, with larger and more consistent increases for foliar Zn applications compared to soil-applied Zn (20–22). However, the increase in grain Zn concentration does not reach to the excess level in a variety of soil types (23). While Zn fertilizers in the form of ZnSO_4_.7H_2_O through soil application (5–25 kg ha^–1^) have been shown to improve grain yield, substantial improvement in grain Zn concentration is achieved mainly *via* foliar sprays (11, 20, 22, 24). The effect of soil application on grain Zn concentration is limited due to fixation in the soil and geochemical factors affecting uptake to plants, while there may be residual effects on yield and grain concentration of up to 10 years (20, 25). The practice of ZAB through soil application could be delivered through the existing soil fertilizer schemes, unlike foliar applications that require additional labor and equipment costs (26). However, the possible integration of pesticide application with foliar fertilization could minimize the cost and facilitate the adoption of foliar application by famers (27). Previous studies employed a more practical approach through combined foliar Zn and pesticide applications (22, 27, 28), which could improve efficiency.

A limited number of studies have tested the efficacy and effectiveness of Zn agronomically biofortified cereal flours for improving human Zn status (29–31). While dietary Zn intakes were increased, there is mixed evidence on the effect on biomarkers of Zn status. These findings are not necessarily contradictory since there is a lack of reliable and responsive Zn biomarkers (32).

In Ethiopia, maize (*Zea mays* L.), teff (*Eragrostis tef* (Zuccagni) Trotter), and wheat (*Triticum aestivum* L.) are the major staple food crops, covering more than 90% of the cropping area and production (33), and providing 70% of the calories in the diet (34). More than 90% of the fertilizer consumption by smallholder farmers is for cereal crops, where almost all (86%) is used for maize (29%), teff (32%), and wheat (25%) (35). Hence, ZAB of staple cereals could be a potential strategy to tackle ZnD in developing countries (36).

The ZAB of staple crops could increase grain Zn concentration and may lead to increased dietary Zn intake in humans, which may lead to a reduction in the burden of disease attributable to ZnD (37). The standard metric of the burden of disease is the disability-adjusted life-year (DALY), a composite indicator of morbidity and mortality associated with a specific health condition (38). It is used to quantify the burden of diseases, including micronutrient deficiencies, to better inform policy decisions (39, 40). Cost-effectiveness analysis (CEA) is a type of economic analysis commonly used to compare the health benefits of a particular intervention with the cost of saving one DALY due to a health problem (40). Quantifying the cost per DALY saved from Zn agronomic biofortification allows evaluation of the effectiveness of an intervention and comparison to alternative interventions (41).

ZAB has been tested in different settings (42–44) on a small scale but with limited economic analysis in low-income settings. For example, a study from China indicated that ZAB of wheat could reduce the burden of ZnD by 57% at a cost of US$226–594 per DALY saved (27). However, such economic analysis is lacking for ZAB in sub-Saharan Africa, which is critical to understanding the cost-effectiveness of ZAB (16, 37) and making informed policy decisions (45). The effectiveness of ZAB could be challenged by several factors, including the cost of the intervention and the availability of infrastructure to disseminate it. It also incurs additional costs, which can be offset by increased yield or health benefits from increased grain Zn content for human consumption (46). However, evidence on the existing disease burden due to ZnD and the potential cost-effectiveness of Zn fertilizer in alleviating disease burden due to ZnD is poorly understood in Ethiopia, where such country-specific evidence is critical (45). The objectives of this paper are therefore to (a) quantify the current existing burden of ZnD in Ethiopia and (b) calculate the potential impact and cost-effectiveness of ZAB of staple crops (maize, teff, and wheat) through granular or foliar Zn application in Ethiopia.

## Materials and methods

### Overview of the study

This study is an *ex-ante* assessment of the burden of disease due to ZnD and the potential health benefits of ZAB of staple cereals (maize, teff, and wheat) through granular and foliar Zn fertilizer applications in Ethiopia. It is based on various sources of secondary information compiled to quantify the existing burden of ZnD, and the potential benefits and costs of a ZAB program. The cost-effectiveness of ZAB of staple cereals in Ethiopia was calculated using a DALYs framework under optimistic and pessimistic scenarios.

### Estimating risk of dietary zinc deficiencies

We used the *per capita* 92-food item food supply data from the recent Food and Agricultural Organization (FAO) food balance sheet (2019) (47) with the Zn and Phytate composition data compiled by Wessells et al. (48) (Supplementary Table 1), to estimate the baseline dietary Zn supplies and estimate of dietary Zn deficiencies among children in Ethiopia. We estimated the absorbable Zn supply using the Miller equation (49), considering the Zn and Phytate molar equivalents after processing. The risk of inadequate Zn intake is estimated assuming normal distribution, where the calculated *Z*-score is translated into the corresponding proportions by comparing the estimated average requirement and the estimated absorbable Zn supplies (48, 50).

### Disability-adjusted life years estimation model

The DALY framework was applied to quantify the baseline burden of ZnD and the potential benefits of agronomically fortified staples crops, considering children as a target population where ZnD is linked to adverse functional outcomes (37). Total DALYs lost due to ZnD were calculated as the sum of years of life lost (YLL) and years lived with disability (YLD) due to diarrhea, pneumonia, and stunting attributable to ZnD as indicated in Equation 1.


(1) $$DALY_{\mathrm{lost}}=\sum_{i=1}^{n}T_{i}M_{i}\left(\frac{1-e^{-\mathrm{rLi}}}{r}\right)+\sum_{i=1}^{n}T_{i}I_{\mathrm{hi}}D_{\mathrm{hi}}\left(\frac{1-e^{-\mathrm{rdhi}}}{r}\right)$$

where: *T_*i*_* = total number of people in target group *i*; M*_*i*_* = mortality rate associated with the deficiency in target group *i, r* = discount rate for future life years; *L*_*i*_ = average remaining life expectancy for target group *i*; I*_*hi*_* = incidence rate of functional outcome *h* in target group *i*; *D*_*hi*_ = disability weight for functional outcome *h* in target group *i, d_*hi*_* = duration of functional outcome *h* in target group *i*.

### Parameters used in baseline disability-adjusted life years estimations

To quantify the baseline burden of ZnD, demographic, epidemiologic, and mortality data were compiled from the literature to fill in the parameters in the equation. We considered diarrhea, pneumonia, stunting, and under-five mortality as functional outcomes of ZnD, where the country-specific incidence and duration of diarrhea (51, 52), pneumonia (53), stunting, and child mortality (54) were compiled from existing literature. Evidence showed that ZnD contributes up to 4.4% of under-five mortality, increases the risk of pneumonia (RR = 1.25; 95 CI: 1.09–1.43) by 25% and diarrhea (RR = 1.09; 95% CI: 1.01–1.18) by 9% among children (3). Furthermore, stunting and ZnD are strongly linked, where a high prevalence of stunting is a proxy for imminent ZnD (55). For mortality and stunting, considered as permanent conditions, the standard life table for Ethiopia (56) was used to calculate YLL or YLD. The total DALYs lost were the sum of YLL and YLD.

In addition, the population statistics and the demographic and health survey 2019 child mortality statistics were compiled by age groups. The age-specific size of the target group was compiled from the updated population estimates (57). The specific mortality indicators for infants (47 deaths per 1,000 births), children aged 1–4 years (12 deaths per 1,000) and under-fives (59 deaths per 1,000 births) were obtained from the recent demographic and health survey (58). We used the percentage contribution of ZnD to childhood morbidities along with the incidence of childhood illnesses to come up with the incidence attributable to ZnD (Table 1). The remaining years lived with disability and disability weights for each outcome were compiled from the global burden of disease (59), with 3% age discounting applied to account for age-related productivity losses recommended by the WHO (37, 60).

**TABLE 1:** Calculated DALY lost attributable to ZnD under optimistic and pessimistic scenarios among children in Ethiopia.

Outcomes of ZnD	Age group	Incidence^a^	Disability weight	Average duration (years)^b^	DALY lost^c^		
					Baseline	Optimistic^d^	Pessimistic^e^
Diarrhea	Infants	0.0139	0.1888	0.0153	170	13	478
	Children 1–4	0.0103	0.1888	0.0153	470	35	1,320
	Children 4 and 5	0.0248	0.1888	0.0153	528	39	1,482
Pneumonia	Infants	0.0725	0.300	0.0110	1,015	731	1,746
	Children 1–4	0.0725	0.200	0.0110	2,523	1,817	4,340
	Children 4 and 5	0.0725	0.200	0.0110			
Stunting	Infants	0.368	0.0010	68.7	45,592	–	–
Mortality	Infants	0.002068	1	68.7	256,205	–	–
	Children 1–4	0.000528	1	70.3	245,613		
Total DALY lost due to ZnD^c^					552,116	550,291	557,023

^a^The incidence rates were calculated based on individual contribution of ZnD to diarrhea (9.9%), pneumonia (25%), and mortality (4.4%) (3) along with the prevalence estimates and ^b^average duration of the diseases among children (51, 52), in Ethiopia. ^c^Refers to the DALY lost due to ZnD under with optimistic^d^ and pessimistic scenarios^e^.

### Data sources for impacts of zinc agronomic biofortification of staple crops

Data on baseline grain Zn concentration, crop fertilizer response, new grain Zn concentration, target crop consumption, bioavailability of Zn, technology coverage, target population size, and other epidemiological data were obtained from different sources (61, 62). The baseline grain Zn concentration of maize (19.1 mg kg^–1^), teff (28.2 mg kg^–1^), and wheat (26.1 mg kg^–1^) was obtained from the recent national GeoNutrition crop survey (*n* = 1,352), covering the three agrarian regions in Ethiopia (63, 64). The response of grain Zn concentration to soil Zn fertilizer application of 19% (maize) and 23% (wheat and teff), and the respective values for foliar Zn application of 30% (maize) and 63% (wheat and maize), were based on results from a metanalysis of field experiments conducted globally (26). Based on the fertilizer response by crop and the method of Zn fertilizer application, the increase in grain Zn concentration was estimated considering the average daily cereal consumption compiled above (Supplementary Table 2). The daily average cereal consumption was obtained from the 2018 Living Standard and Measurement Survey (65), which collected relevant data on cereal consumption in a nationally representative sample. The food consumption was collected at the household level over a 7-day recall period under a standard protocol. The survey metadata has a standard region-specific conversion factor to convert local units of consumption in to kilogram equivalents for each measurement unit to allow us to estimate the standard consumption amount in grams *per* child equivalent. Thus, cereal (maize, teff, and wheat) consumption was converted into g *per capita per* day, and this was used to estimate the baseline and improved Zn intake from each cereal.

Then, the total absorbable dietary Zn supply with improved crop composition was calculated based on the Zn bioavailability and Zn fertilizer coverage under optimistic and pessimistic scenarios. We assumed that 24% of Zn was bioavailable under whole grain consumption (high phytate to Zn molar ratio), where its bioavailability is limited (66).

To capture the various sources of uncertainty, exist in estimating technology coverage (Zn fertilizer application), we made alternative assumptions under optimistic and pessimistic scenarios to obtain a more reliable estimate. The current soil fertilizer application coverage was calculated from the recent national Agricultural Sample Survey (AgSS) by crop (55–75%) (35). Hence, we calculated the DALY saved through soil Zn fertilizer coverage at a scale of 75% under optimistic and 55% under pessimistic scenarios considering the current fertilizer application by crops (Supplementary Table 2).

ZAB of staple crops *via* foliar sprays is a more effective strategy than *via* soil application to increase grain Zn concentration (11). We conducted the cost-effectiveness analysis under two scenarios: isolated foliar Zn application and through combined application with pesticide application. Joy et al. (26) conducted a cost-effectiveness analysis for ZAB in sub-Saharan Africa, where adoption of foliar Zn fertilizer was calculated under scenarios of 50 or 75% of cropland. For comparability, we considered the same scenarios of adoption rates to estimate the DALYs saved and costs. Isolated ZAB of crops *via* foliar application would be costly due to repeated applications and some extra expenses (67). However, studies indicated that the cost associated with ZAB through foliar application could be substantially reduced by integrating it with regular pesticide applications (22, 27). Considering the level of existing pesticide use by crops (16.4–61% of cropping area), a single combined foliar Zn application with pesticide was assumed. We also conducted the analysis under different coverage rates to decide on the minimum coverage where the intervention could be cost-effective.

Consumption of ZAB crops could increase human Zn intakes and reduce health outcomes related to ZnD. Based on the improved grain Zn concentrations in response to Zn fertilizer, the anticipated new Zn intake (*BI)* was calculated. We assumed that soil Zn fertilizer application led to an average increase in grain Zn concentration of 23% in maize and 19% in teff and wheat, while increases of 30% in maize and 63% for teff and wheat were assumed following foliar Zn fertilization. This increase in grain Zn concentration was taken from Joy et al., obtained from a metanalysis of field experiments (26). Based on the fertilizer response and the increase in total dietary Zn intake through biofortified grain consumption, and compared against the RDA for Zn. Hence, we considered the RDA for Zn for infants (7.5 mg day^–1^) and older children (8.3 mg day^–1^), under a low bioavailability and higher phytate to Zn ratio (66). Finally, the estimated efficiency of ZAB was calculated and translated into the impact of ZAB as detailed above.

### Change in prevalence of zinc deficiency due to zinc agronomic biofortification of staple crops

The potential change in the burden of ZnD with the consumption of ZAB staple crops was estimated by calculating the efficiency (*e*) of agronomically biofortified cereal consumption as stated in **Equation 2**. The efficiency is calculated by comparing the improved Zn intake after intervention (BI) to the baseline Zn intake before intervention (CI) against the recommended dietary allowance (RDA) (37, 68). The RDA is the level of intake that meets the requirements of 97.5% of a specific demographic group.


(2) $$e=\frac{\ln\left(\frac{\text{BI}}{\text{CI}}\right)-\ln\left(\frac{\mathrm{BI}-\mathrm{CI}}{\text{BI}}\right)}{\ln\left(\frac{\text{RDA}}{\text{CI}}\right)-\ln\left(\frac{\mathrm{RDA}-\mathrm{CI}}{\text{RDA}}\right)}$$

where:

*e*: efficiency of the intervention, ZAB

BI: Zn intake level (mg capita^–1^ day^–1^) after the intervention, ZAB

CI: current Zn intake in mg capita^–1^ day^–1^

RDA: the recommended dietary allowance for Zn for specific age group from WHO (66).

The dietary Zn intake from grain (*CI)* was estimated based on the baseline grain Zn concentration and *per capita* cereal consumption from a recent national socioeconomic survey compiled by the World Bank (69). Dietary Zn intake from agronomically biofortified zinc (*BI)* was calculated based on the baseline grain composition, the estimated response to the Zn fertilizer, and typical *per capita* cereal consumption converted to infant and child equivalents. The proportional decrease in the incidence of ZnD-associated morbidity and mortality indicators was calculated by the efficiency rate *e* (Equation 2), and this was used to calculate the new DALY burden with ZAB grain consumption. Then, the DALY that could be averted through ZAB was calculated by comparing the baseline and the new DALY burden with biofortified grain consumption and expressed as a percentage of the DALY burden that could be averted through ZAB.

### Cost-effectiveness analysis

#### Cost estimations

The cost of Zn fertilizer application was estimated from the existing market prices, including the fertilizer, labor, and equipment costs and we did not consider extension costs. For the foliar Zn fertilizer application, labor and material costs are included, but not for the granular application, as the Zn can be blended into the existing fertilizers without a need in change of practice by the farmer. For each crop, we made assumptions on the potential Zn fertilizer coverage of 55–75% for soil application based on national agricultural statistics (35), and optimistic and pessimistic scenarios of 75 and 50% coverage for foliar application to estimate the total costs (26).

The total fertilizer requirement and costs are calculated based on the price of Zn fertilizer of US$ 0.78 per kg of Zn fertilizer, considering the transport and other related costs before it reaches the farmers (70, 71). The total fertilizer cost for granular and foliar Zn was calculated considering the cropping area, the anticipated coverage (50–75%), the unit price per kg of fertilizer and the possible application rate (Supplementary Table 2). Fertilizer application at a rate of 25 kg Zn ha^–1^ for granular and 12.5 kg Zn ha^–1^ for foliar fertilizer application was assumed. Zinc fertilizer in the form of zinc sulfate provide a more bioavailable Zn for crops through cost effective means (20). Since, ZnSO_4_.7H_2_O contain 22% Zn content, where optimal application rate of 25 kg ZnSO_4_.7H_2_O ha^–1^ could provide adequate Zn in crops of 5.5 kg Zn ha^–1^ (4–6 kg Zn ha^–1^) (72, 73). In addition, scaling up foliar Zn fertilizers at the level of current pesticide use was simulated and presented in the result. We calculated that 29% of the cropping area is treated with pesticides, where 16% (maize), 54% (teff), and 61.2% (wheat) of the crops are treated with pesticides in Ethiopia (35).

A knapsack sprayer was assumed to cost US$51, which may be used for 10 ha with a unit price of US$5.1 ha^–1^ year^–1^, where the total sprayer cost was estimated in proportion to the cropping area under optimistic and pessimistic scenarios explained above. Based on a two-times-per-year foliar application (1.5 hectares could be covered by one person) (27), the annual labor cost per ha will be US$5.3. Similarly, the total labor cost for ZAB was estimated in proportion to the total cropping area covered by each crop considering the different fertilizer coverages. In addition, integrated foliar Zn application to the level of existing pesticide application was also considered. Hence, we assumed that the labor cost could be omitted and no extra material cost (for knapsack) would not be needed, as the majority of smallholder farmers mainly rely on their own labor sources for agricultural productivity. In that case, we assume zinc can be added to the pesticide or applied separately using existing pesticide application resources, and would not require additional costs (Supplementary Table 3).

#### Cost-effectiveness evaluation

All the costs associated with fertilizer, material, and labor costs for each crop were juxtaposed to the total DALYs averted through ZAB under different scenarios to calculate the cost of saving one DALY. Based on the WHO (74) recommendation, a cost-effective intervention may be defined as one that saves a DALY at 1–3 times the national GDP per person (74). Interventions that save a DALY at a cost less than the GDP per capita are regarded as highly cost-effective (74, 75). We considered the World Bank GDP *per capita* of US$936 for Ethiopia and we valued the same for each DALY lost due to a health problem (ZnD) (76).

## Results

### Estimates of dietary zinc supply and risks of dietary zinc deficiency

The result of the compiled FAO food balance sheet and nutrient composition data found that the baseline national total dietary Zn supply was 5.13 mg day^–1^ while the potentially absorbable Zn supply was 3.42 mg day^–1^ for children. An estimated 49% of children had a risk of inadequate Zn intake below the age-weighted estimated average requirement (7.9 mg day^–1^). Among the commonly consumed food items, an estimated 30% (1.56 mg), 19.5% (1.0 mg) and 6.5% (0.34 mg) of the total dietary Zn supply were from other cereals, mainly from teff, maize and wheat products, respectively. Similarly, the *per capita* absorbable Zn supplies of 3.42 mg *capita*^–1^ day^–1^ were supplied from other cereals, including teff (29.0%), maize (18.3%), barley (9.8%), and wheat products (6.9%) (Supplementary Table 1).

### Baseline burden of zinc deficiency among children

The total DALY lost due to ZnD among under-five children is presented in Table 1 under optimistic and pessimistic scenarios. The calculations lead to an estimated 0.55 million DALYs are attributable to the health consequences of ZnD in Ethiopia, 91% of which from child mortality. Sensitivity analysis indicates an estimated 550,291 DALYs lost under an optimistic scenario and 557,023 DALYs for the pessimistic scenarios (Table 1).

### Improved dietary zinc supply through zinc agronomic biofortification

The estimated dietary Zn intake could be improved through consumption of crops grown under ZAB through foliar and granular Zn fertilizers among children. For instance, the dietary Zn intake could be increased by about 14% from the baseline intake level of 1.74–2.0 mg *capita*^–1^ day^–1^. Similarly, the dietary intakes from each cereal could be increased proportionally, where the increase was higher for foliar Zn fertilizers compared to granular applications. The efficacy in increasing the dietary Zn intake was higher with foliar application (*e* = 0.288). Furthermore, the increase in grain Zn concentration after ZAB was low (23–30%) for maize compared to teff and wheat (19–63%) through granular and foliar Zn fertilizers, respectively (Supplementary Table 4).

### Potential cost-effectiveness of zinc fertilizers

The corresponding DALYs saved and the potential cost-effectiveness (US$ to save one DALY) are presented in Table 2. An estimated 29–38% of DALYs due to ZnD could be averted at a cost of US$502 and US$505 *via* granular ZAB of staple crops under optimistic and pessimistic scenarios, respectively. ZAB through granular Zn fertilizers could be more cost-effective in the teff crop than in wheat and maize. Implementation at a scale of 75%, granular Zn fertilizer could be cost-effective at an expenditure of 419–424 US$ per DALY saved and 413–423 US$ per DALY saved by teff and wheat ZAB, respectively. In contrast to granular Zn application, ZAB through foliar application is more cost-effective, reducing the burden of human ZnD by 32% under pessimistic and 40% under optimistic scenarios. When disaggregated by crop, ZAB of maize, teff, and wheat had the potential to reduce the burden of ZnD by 23, 8.5, and 8.6%, respectively. While ZAB through foliar Zn fertilizer could cost of US$226–496 to avert one DALY due to ZnD. Foliar Zn application for teff was found to be more cost-effective, costing US$ of 153–341 to save one DALY than maize (US$389–817) and wheat (US$278–603 per DALY saved, Table 2).

**TABLE 2:** DALYs saved and cost-effectiveness of ZAB through granular and foliar Zn fertilizer in Ethiopia under “optimistic” and “pessimistic” scenarios of adoption rates.

	DALYs saved and % reduction				Cost (US$) per DALY saved			
Granular Zn application	Optimistic		Pessimistic		Optimistic^c^	Pessimistic^d^		
		%		%				
Maize	46,277	8.4	32,757	5.9	798	827	–	–
Teff	100,950	18	74,926	14	419	424	–	–
Wheat	65,603	11.9	49,234	8.9	413	423	–	–
Overall^a^	212,830	38	156,917	29	502	505	–	–

**Foliar Zn application**					**e**	**f**	**g**	**h**

Maize	46,736	8.5	35,995	6.5	389	817	287	602
Teff	129,629	23	106,321	19	153	341	112	252
Wheat	47,535	8.6	37,828	6.9	278	603	205	445
Overall^b^	223,900	40	180,144	32	226	496	167	366

^a^Based on the soil application of Zn fertilizer at rate of 25 kg per ha^-1^ and ^b^foliar Zn fertilizer application (12.5 kg per ha^-1^) to the three staple crops (maize, teff, and wheat), Soil Zn fertilizer application at coverage of 55%^c^ and 75%. ^d^Of the crop area based on the CSA, 2021 report; foliar Zn fertilizer application considering 50%^e^ and 75%^f^ technology coverage [assumption from (26)], respectively. The cost per DALY saved through foliar Zn fertilizer excluding the labor cost at 50%^g^ and 75%^h^ of the cropping area.

Fertilization of staple crops *via* foliar Zn fertilizers could be a cost-effective strategy to decrease the burden of ZnD at a cost of US$167 under optimistic and US$366 pessimistic scenarios to save one DALY. Similarly, the use of foliar Zn fertilizer for maize (US$287–602 per DALY) and wheat (US$205–445 per DALY averted) could be marginally cost effective. Teff foliar fertilization could be more effective at US$112–212 per DALY averted, which is below the national GDP and hence the WHO and the World Bank threshold for cost-effective public health interventions (Table 2).

In the other scenario, we assume ZAB of staple crops with foliar Zn was combined with pesticide application, which reduces the material and labor costs for ZAB. Scaling up ZAB to the level of existing pesticide application to staple crops could reduce the burden of ZnD by 32.6% and be cost-effective at a cost of US$260–353 per DALY saved. ZAB to maize, teff, and wheat contribute to 19.3, 6.9, and 6.5% reduction in ZnD burden, respectively. Moreover, ZAB of teff *via* combined foliar and pesticide application would be more cost-effective at cost of US$221–300 per DALY saved (Table 3).

**TABLE 3:** Potential cost-effectiveness of the use foliar Zn fertilizers combined with pesticides, considering the existing pesticide coverage by crops in Ethiopia^a^.

Crop	DALY saved (% reduction)	Cost-effectiveness (US$ per DALY saved)	
		Optimistic^b^	Pessimistic^c^
Maize	35,832 (6.5%)	172	233
Teff	106, 363 (19.3%)	221	300
Wheat	37,875 (6.9%)	455	618
Overall	180,070 (32.6%)	260	353

^a^We considered pesticide coverage of 16.4%, 54% and 61.2 for maize, teff, and wheat based on the CSA report on fertilizer and pesticide use by crop type in 2021 ^b^excluding the labor cost and ^c^including the labor cost for foliar application.

## Discussion

The predominance of cereal-based diets with a low grain Zn concentration and low bioavailability of Zn is a major factor underlying widespread human ZnD (20), where low grain Zn concentrations are partially caused by low concentrations of plant-available Zn in soils and low soil-to-crop transfers. It is also indicated that the soil Zn concentration is low (below 0.5 mg kg^–1^) or marginal in the major agricultural areas of the country (77). Compared to other micronutrient deficiencies (vitamin A deficiency, iron deficiency and iodine deficiency) with an established cost-effective strategy, ZnD in Ethiopia needs a feasible and cost-effective strategy besides Zn supplementation (16). ZAB of staple crops could play a role in alleviating ZnD (20, 24), with ZAB shown to improve grain Zn concentrations (26), leading to increased dietary Zn consumption (26, 78). Hence, we evaluated and quantified the baseline burden of ZnD and the potential cost-effectiveness of ZAB for staple crops in Ethiopia.

We estimated that human ZnD contributes to 0.55 million DALYs lost attributable to ZnD-related childhood morbidity and mortality. Most of the ZnD burden is linked to its contribution to overall child mortality and stunting. This huge burden comes from the fact that diarrhea and respiratory infections (including pneumonia) are the top five causes of morbidity and mortality in the country (79). The currently estimated burden of ZnD is very close to Joy et al.’s estimate, which estimates 0.4 million DALYs annually are lost due to human ZnD (26). For comparison, in Ethiopia an estimated 0.45, 0.4, and 0.09 million DALYs annually are lost due to iron, vitamin A, and iodine deficiencies, respectively (80). A recent national survey using Serum Zn also indicated that 72% were ZnD (8) and up to 50% had low dietary Zn intakes (81), comparable with the current estimate that more than 49% of children had inadequate dietary Zn intake. This indicates that ZnD is a major public health problem significantly contributing to morbidity and mortality outcomes in Ethiopia.

Our results show that ZAB of maize, teff, and wheat through soil Zn fertilizer application could reduce the burden of ZnD by 29% under a pessimistic uptake scenario to 38% under an optimistic uptake scenario, at a cost of US$502–505 per DALY saved. Out of the three crops, teff is the most effective at reducing the burden of ZnD by 14–18% compared to wheat (8.9–11.9%) and maize (5.9–8.4%). This is due to the greater response to Zn fertilizer application of grain Zn concentration in teff compared to wheat or maize. The use of enriched soil fertilizer is the most convenient and would likely lead to the highest adoption, as no extra labor or extension costs would be needed. However, soil application is limited in that adsorption of Zn to soil particles might limit Zn uptake by crops and, ultimately, for human consumption. Moreover, soil application uses a larger quantity of Zn per hectare compared to foliar application, which has cost implications. Hence, to maximize a ZAB policy will require spatially informed Zn fertilizer recommendations based on soil factors such as clay content, soil pH, organic matter, and other geochemical factors that influence the effectiveness of soil Zn application (82). Beyond the increase in grain Zn concentration, application of Zn fertilizers may improve the yield of maize and wheat and could contribute to an improvement in food security for the rural poor. This could contribute to lower ZnD due to malnutrition-related morbidity and ZnD. Previous studies have shown that potential yield benefits may outweigh health benefits through averted ZnD, on a monetary basis (46).

The response of crops to Zn fertilizer depends on soil factors (20) which in turn affect the DALYs averted through ZAB. A study from Ethiopia indicated a large proportion of children (4 million) reside in soils with marginal ZnD (1.5–2.5 mg kg^–1^ Zn), which cover large parts of the crop production area in comparison to the relatively small area of Zn-deficient soils (0.3 million children). Therefore, ZAB on marginally Zn-deficient soils could potentially improve the grain Zn concentration for a larger number of people and impact the burden of ZnD significantly more than on Zn-deficient soils (77). This emphasizes the need for geographically targeted fertilizer use for maximum public health impact.

On the other hand, the use of Zn fertilizer on marginally deficient soils, while having a positive effect on grain Zn for maize and wheat (83), may only have a limited effect on crop yield, which may discourage smallholder farmers from adopting such interventions. However, farmers could benefit through consumption of improved grains with a better Zn content for better health. The existing fertilizer subsidy program could be leveraged to support cost-effective schemes for the large-scale use of Zn-enriched fertilizers (84), which can be compensated by the health benefits with a limited gain in yield. In addition, the availability of Zn-enriched cereals on the market might positively contribute to a reduction in the burden of ZnD beyond producers and impact the burden of ZnD at large. However, the practice of industrial and some traditional processes might cause significant Zn loss and reduce the efficacy of Zn fertilizers in reducing the burden of ZnD. While some traditional processing methods, primarily fermentation (maize, teff, and wheat crops used to make “injera,” breads, and other products), may reduce phytate content and increase Zn bioavailability for human consumption (85), the habit of whole-grain consumption (with high phytate content) (86) might limit Zn absorption. Meanwhile, foliar Zn application showed to increase the whole grain Zn (by 58%) and flour Zn (by 76%) and tends to reduce the phytate to Zn molar ratio (87), which might also improve bioavailability and contribute to a lower burden of ZnD.

Foliar Zn fertilizers are more effective at increasing grain Zn in wheat (22) and maize (20) as compared to soil Zn application, which could improve Zn nutrition and contribute to a reduced burden of disease due to ZnD. Our analysis indicated that foliar Zn fertilizer application at a scale of 50 and 75% of the cropping area would cost US$167–366 per DALY saved. Potential cost-effectiveness of ZAB of wheat has been indicated in China (26) and Pakistan (46). ZAB *via* foliar application can be a more cost-effective strategy to tackle grain and human ZnD due to the greater response of grain Zn concentration to foliar vs. soil-applied Zn (26). A study by Wang et al. also showed that ZAB could improve Zn intake and reduce the burden of disease due to ZnD by 57% (27). A case study from Pakistan also showed that the use of Zn fertilizer on wheat could reduce ZnD at a cost of US$461–619 (46).

Combining foliar Zn and pesticide application could reduce the cost of ZAB, as they take out the labor and equipment costs. In this study, combined applications were found to be more cost-effective, with a cost of US$124–217 per DALY saved. A similar study from China showed that combined foliar Zn and pesticide application in wheat is more cost-effective (US$41–108 per DALY saved) through combined foliar and pesticide application (27). Another study from China, this time on rice, also indicated that combined Zn fertilizer and pesticide application is cost-effective, at a cost of US$38–530 per DALY saved, because of the reduction in labor and material costs (88). However, integrating the pesticide application with foliar Zn application could be challenging and would require farmers’ willingness.

The selection of crops for Zn fertilization should consider the response and effectiveness of these crops. For instance, the use of soil and foliar Zn fertilizers on teff could save a greater burden of disease due to ZnD than wheat and maize. This is partly explained by the larger response in grain Zn concentration in teff compared to wheat and maize following Zn fertilizer application (63). Also, traditional processing of teff involves partial fermentation, which degrades phytic acid and improves bioavailability, and its products are consumed by a large segment of the population (89). Beyond that, the differential uptake of soil and foliar Zn biofortification could make a difference in the potential impact of different Zn fertilizer applications. Wheat has been shown to have significantly higher Zn uptake and leaf-to-grain translocation of Zn than maize, regardless of the application method (90). Besides agronomic approaches, biofortification through plant breeding might be more effective, with a previous study estimating a cost of US$11–146 per DALY saved due to ZnD (75), which is lower than the current estimates for ZAB.

However, for biofortified varieties to reach their genetic potential in terms of grain Zn accumulation, which still requires that the plant can access sufficient Zn. Under Zn-limited conditions, biofortified varieties may fail to deliver more Zn (19), and further research is required to understand the role of Zn fertilizers used in tandem with genetically biofortified varieties. However, problems with farmer acceptance and the need for large-scale investment could make large-scale use of such interventions less feasible (45, 91), where our analysis indicated that ZAB is a cost-effective intervention to reduce ZnD at an acceptable cost. It would be a cost-effective strategy to implement combined granular and foliar Zn fertilizers as a short-term measure to reduce ZnD in the country. Furthermore, to our knowledge, Zn-biofortified teff varieties are not under development, so ZAB provides an opportunity to increase dietary Zn supplies in a shorter timeframe.

### Limitations of the study

This study employs multiple assumptions, and the evidence-base on the potential effectiveness of ZAB remains relatively weak. Nonetheless, ex-ante studies remain useful as a way to generate approximate values of cost-effectiveness for comparison with other potential investments. Zinc fertilizer coverage is particularly difficult to estimate accurately, as it depends heavily on the mode of scale-up pursued, and there might be practical barriers to scale up which are difficult to ascertain. We tried to capture some of this uncertainty using optimistic and pessimistic scenarios of uptake, but even this range may be too high. Moreover, the crop response to Zn fertilizers is likely to vary according to many factors, including soil geochemistry, landscape position, farmer practices, etc. which will affect the dietary intake and effectiveness of ZAB at a large scale, potentially with important spatial variation. Due to the absence of agronomic field trials and considering the existing fertilizer use pattern, the analysis is limited to the three major staple cereals (maize, teff, and wheat). However, there are districts or regions where some minor cereals (sorghum and barley) predominate and might have a potential for ZAB.

## Conclusion and recommendations

In Ethiopia, the use of granular and foliar Zn fertilizers on maize, teff, and wheat could save a substantial part of the burden of disease due to ZnD. It could be a potentially cost-effective strategy to address ZnD and contribute to alleviating food insecurity in Ethiopia. Inclusion of yield effects following soil-Zn applications might further increase benefit-to-cost ratios, while adding Zn to pesticide sprays could minimize additional costs and increase cost-effectiveness. It appears more cost-effective to apply Zn to teff and wheat compared to maize, and a strategy to scale-up granular Zn fertilizers could operate through the input subsidy program. There is a need for further evidence on the geographic targeting of Zn fertilizers for maximum impact and to evaluate the potential policy directions to introduce Zn fertilization of staple crops.

## Data availability statement

The original contributions presented in this study are included in the article/Supplementary material, further inquiries can be directed to the corresponding author.

## Ethics statement

The studies involving human participants were reviewed and approved by the University of Nottingham, School of Sociology and Social Policy Research Ethics Review Committee (BIO-1718-0004) and the Addis Ababa University, College of Natural and Computational Sciences, Institutional Review Board Committee (CNCSDO/187/14/2021). Written informed consent for participation was not required for this study in accordance with the national legislation and the institutional requirements.

## Author contributions

AA, HD, EJ, DK, MB, and DG: conceptualization and designing the study. AA, DG, HD, and MB: acquisition of data. AA: organizing preliminary data and statistical analysis, interpretation of data, and preparing, writing, and drafting the manuscript. AA and EJ: supplementary material design and preparation. All authors: review, editing, read, and approve the final manuscript.

## Abbreviations

AgSS: Agricultural Sample Survey
CI: Current Intake
BI: Biofortified intake level
DALY: “Disability Adjusted Life Years Lost”
GDP: Gross Domestic Product
ha: hectare
Kg: kilogram
RDA: Recommended Dietary Allowance
WHO: World Health Organization
ZAB: Zinc Agronomic Biofortification
Zn: Zinc
ZnD: Zinc Deficiency
ZnSO_4_: Zinc Sulfate.
